# Low 25OH Vitamin D Blood Levels Are Independently Associated With Higher Amyotrophic Lateral Sclerosis Severity Scores: Results From a Prospective Study

**DOI:** 10.3389/fneur.2020.00363

**Published:** 2020-05-29

**Authors:** Raul Juntas-Morales, Nicolas Pageot, Gregory Marin, Anne-Marie Dupuy, Sébastien Alphandery, Laura Labar, Florence Esselin, Marie Christine Picot, William Camu

**Affiliations:** ^1^Clinique du Motoneurone, Explorations Neurologiques, CHU Gui de Chauliac, Montpellier, France; ^2^Département d'Information Médicale, CHU de la Colombière, Montpellier, France; ^3^Laboratoire de Biochimie et Hormonologie, CHU Lapeyronie, Montpellier, France

**Keywords:** amyotrophic lateral sclerosis, vitamin D, prognosis, multivariate analysis, prospective study

## Abstract

**Background:** Amyotrophic lateral sclerosis (ALS) is a fatal neurodegenerative disorder characterized by progressive degeneration of upper and lower motor neurons. Prognosis is highly variable, ranging from few months to more than 30 years. 25OH vitamin D (25OH VD) blood levels have been associated with worse prognosis of ALS, but these results remain in dispute. We addressed this controversy with a prospective study and multivariate analysis to study the influence of known clinical prognostic factors of the disease and 25OH VD levels on Revised Amyotrophic Lateral Sclerosis Functional Rating Scale (ALSFRS-R) severity score (ALS-SS), as defined by the monthly rate of decline of ALSFRS-R score, to identify the factors most closely linked to the risk of worsening of the disease.

**Results:**This prospective cohort of ALS patients recruited 127 individuals, and 105 of them met inclusion criteria. Mean age of onset was 62.2 ± 12.1 years, 32% of subjects had bulbar onset, and gender ratio was 1.44 (male/female). Mean 25OH VD level was 26.8 ± 10.8 ng/ml and was similar between males and females. Patients with 25OH VD levels <15 ng/ml had significantly higher ALS-SS at inclusion (ALS-SSi) than those with normal levels (>30 ng/ml), *p* = 0.011. The study of ALS-SS as calculated at the end of follow-up (ALS-SSe) was not found correlated to initial 25OH VD levels (*r* = −0.19; *p* = 0.084). Univariate analysis showed that ALS-SSe correlated with 25OH VD levels, ALS duration at inclusion, slow vital capacity (SVC) at inclusion, and SVC loss. Multivariate model showed that 25OH VD levels were independently associated with ALS-SSe: *r* = −0.0125, *p* = 0.033. Log rank test with Kaplan–Meier curves did not show significant differences of survival between the groups defined by 25OH VD levels: <15, >15 and <30, and > 30 ng/ml, *p* = 0.88.

**Conclusions:** This prospective study in ALS patients confirmed previous retrospective results: ALS-SSi is significantly higher in patients with severe VD deficiency. For the first time, multivariate analysis showed that 25OH VD level was an independent prognostic factor correlated to ALS-SSe, suggesting that discrepancies between previous works could be due to confounders. It would be important that the present work be replicated in larger samples to confirm the present findings.

## Background

Amyotrophic lateral sclerosis (ALS) is a fatal adult-onset neurodegenerative disorder characterized by the progressive degeneration of upper and lower motor neurons. Motor neuron loss causes muscle weakness and atrophy, leading to death in a median time of 3 years (1). However, ALS prognosis is highly variable, ranging from few months to more than 30 years. Some ALS-related conditions have been associated with an increased severity such as age of onset, weight loss, or vital capacity (2). The influence of others factors on prognosis such as depression, smoking, dyslipidemia, or statin treatment remains controversial (2–6). Several biomarkers have also been associated with ALS prognosis such as UNC13A gene polymorphism, blood ferritin levels, blood creatinine levels, serum neurofilament light chain, or 25OH vitamin D (25OH VD) levels; but to date, none of them has been unequivocally linked to ALS onset or progression (2, 4, 7). Particularly, the association between a worse prognosis and VD deficiency has given controversial results. In a retrospective work in 74 ALS cases, VD deficiency was associated with a fourfold higher rate of decline of Revised Amyotrophic Lateral Sclerosis Functional Rating Scale (ALSFRS-R) score (7). Conversely, in a study of 125 patients, a statistical trend toward a worst prognosis in ALS patients with the highest 25OH VD levels was found (8). Others did not find any relationship between 25OH VD levels and ALS prognosis, suggesting that worst prognosis in patients with VD levels was associated with gross motor impairment (9). However, the potential role of VD deficiency in ALS has been reinforced by a series of neurobiological studies. In neuronal cell culture, VD improved motor neuron survival in a dose-dependent manner and completely blocked Fas-ligand-induced cell death (7). A VD_3_-deficient diet delayed disease onset but decreased motor performance of SOD1 mutant mice, and VD intake increased the strength of these mice SOD1 mice (10, 11). These data were consistent with a neuroprotective activity of VD against neuronal damage, prompting the proposal of VD as a potential treatment option for ALS, with some interesting results (12, 13).

Interestingly, VD has been proposed to act as a neuroprotective factor in several neurological disorders or conditions including Parkinson's disease, multiple sclerosis, cognitive troubles, and neurovascular disorders (14–16). Moreover, low plasma levels of 25OH VD have been associated with an overall worse prognosis in those disorders (17–21).

## Aim of The Study

As previous studies of 25OH VD levels in ALS raised several methodological concerns and were apparently discordant, we tried to address those points in a prospective study with a large analysis of known clinical prognostic factors of the disease. Our main objective was to identify the relationships of 25OH VD blood levels with the risk of worsening of ALS as measured by the rate of ALSFRS-R decline adjusting on known clinical prognostic factors (22).

## Methods

### Ethics Statement

The present study was approved by the national ethics committee Sud-Méditerranée IV. All patients gave informed and written consent before entering the study. Once collected, samples were anonymized by the study coordinator, Sébastien Alphandéry, before processing by the biochemistry lab. The study was registered on clinicaltrials.gouv NCT01823380.

### Subjects

All ALS patients were included prospectively and followed up quarterly in the ALS center of Montpellier, according to national recommendations. Inclusion criteria were as follows: (1) a diagnosis of possible, probable, or definite ALS according to Airlie House/El Escorial revised diagnostic criteria, at inclusion, (2, 23) disease onset less than 3 years at inclusion visit; (3) patient aged 18 to 95 years; (4) patient already followed up for at least 6 months in the center before inclusion; and (5) patient able to give informed consent to enter the trial. Exclusion criteria were as follows: (1) being treated with a drug-containing VD, of any type, in the preceding 6 months before inclusion; (2) ALSFRS-R score <20; (3) a diagnosis of dementia; (4) pregnant or breastfeeding woman. Dropout criterion was as follows: a patient was excluded from the study if ALS diagnosis does not fulfill the criteria of probable or definite ALS at the end of follow-up, for example, 1 year, or before death if death occurred before this delay.

### Clinical and Biological Data

For each patient, the following data were systematically collected at inclusion: (1) gender; (2) age of onset, defined as the age at which the first weakness occurred; (3) site of onset, bulbar, upper limb, or lower limb, corresponding to the site at which first symptoms of muscle weakness were noted; and (4) ALS duration since onset, corresponding to the delay between ALS onset and inclusion in the study. The following parameters were collected quarterly: (1) weight, including weight loss at entry by reference to the usual weight; (2) ALSFRS-R score; and (3) slow vital capacity (SVC). For each patient, ALS severity score (ALS-SS) was calculated. ALS-SS corresponded to the rate of decline of the ALSFRS-R score per month (22). Two different ALS-SS were thus obtained: ALS-SS at inclusion, corresponding to the severity score from onset and inclusion (ALS-SSi): and ALS-SS at the last follow-up visit, either 6 months or 1 year (ALS-SSe). ALS duration was also recorded, corresponding to the delay between onset of weakness and death, in months. 25OH VD was determined by the Elecsys® VD assay on a cobas 8000-e602 according to the manufacturer's recommendations with limit of detection (LOD) at 3.00 ng/ml and intra-assay and inter-assay <6.8% and <13.1%, respectively. For each patient, this measure was done at inclusion only and was expressed in ng/ml.

### Statistical Analysis

The baseline characteristics were first reported (*n* = 105). Quantitative variables (age at onset, ALSFRS-R score, weight loss, and SVC 25OH VD) were expressed by their mean ± standard deviation, and the qualitative variable (site of onset) with frequencies and proportions (%) of the different categories.

The association between 25OH VD levels at inclusion and ALS-SSewas was evaluated using the Spearman correlation test. Criteria at inclusion (ALS-SSi, SVC loss, weight loss, and 25OH VD levels) were compared according to site of onset using ANOVA. 25OH VD levels at inclusion were also categorized into three groups (<15, from 15 to 30, and >30 ng/ml) and compared for clinical characteristics at inclusion (age at onset, ALS-SSi, ALS duration at inclusion, SVC, and weight loss) using ANOVA and *post hoc t*-test.

To study the possible impact of several variables of interest on ALS-SSe, univariate linear regression models were carried out in the group of patients for whom ALS-SSe could be calculated (at least 6 months of follow-up, *n* = 77). The variables with a *p*-value lower than 0.15 were then considered for a multivariate model, and the variables with a *p*-value lower than 0.05 in the multivariate model after a stepwise selection of variables were considered statistically significant and were reported in the table.

The relationship between 25OH VD levels and ALS duration was also studied considering the prognostic value of 25OH VD concentrations both as a continuous variable and at the 30 ng/ml threshold and the influence of 25OH VD levels on survival was analyzed using log rank test with Kaplan–Meier curves, comparing patients with 25OH VD levels <15 ng/ml with those with levels between 15 and 30 ng/ml and those with levels > 30 ng/ml.

Statistical analyses were performed using SAS 9.1 software (SAS Institute, Cary, North Carolina), and the statistical bilateral significance threshold was set at 5%.

## Results

### General Characteristics of the Study Population

**Figure 1:**
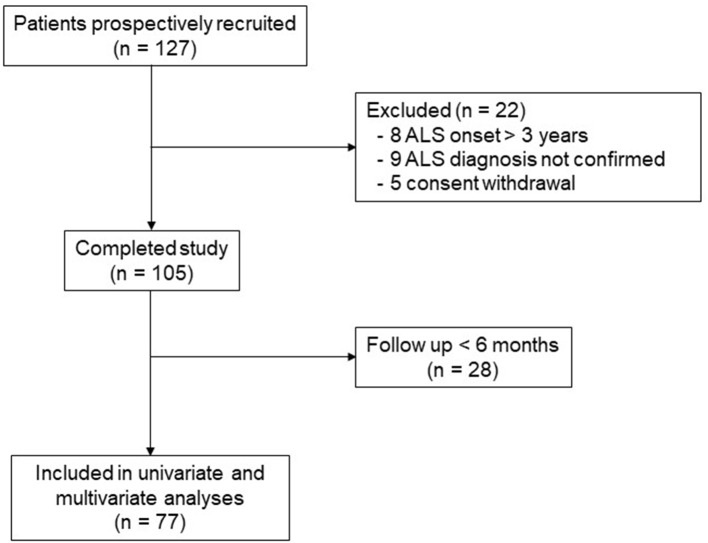
Study flowchart.

A total 127 patients were included in this study, and 22 of them met exclusion or dropout criteria or withdrew consent (Figure 1). For the 105 remaining ALS patients, general characteristics well corresponded to the usual ALS population with age of onset at 62.2 ± 12.1 years, 32% of subjects had bulbar onset, with a slight male predominance with 62 men and 43 females and gender ratio of 1.44 (Table 1). Mean 25OH VD level in the whole population was 26.8 ± 10.8 ng/ml and was similar between males and females. ALS-SSi was higher in bulbar patients, but this difference was not statistically significant (Table 2). There were also some differences between the groups for weight loss and SVC loss at inclusion, but, again, those differences were not statistically significant. Conversely, according to site of onset, 25OH VD levels were quite similar.

**Table 1:** Population characteristics at inclusion.

	**All**	**Males**	**Females**
***N***	**105**	**62**	**43**
Age at onset	62.2 ± 12.1	60.9 ± 13.7	64.1 ± 9.1
Site of onset (*n*)			
Bulbar	32	16	16
Upper limb	36	26	10
Lower limb	37	20	17
ALSFRS-R score	37.7 ± 6.3	37.7 ± 5.7	37.7 ± 7.2
SVC (%)	81.0 ± 22.1	79.8 ± 20.6	82.9 ± 24.3
Weight loss (kg)	0.3 ± 0.6	0.3 ± 0.6	0.2 ± 0.4
25OH vitamin D (ng/ml)	26.8 ± 10.8	27.4 ± 11.2	25.9 ± 10.4

*Value are means ± SD or n*.

*ALSFRS-R, amyotrophic lateral sclerosis functional rating scale revised; SVC, slow vital capacity*.

**Table 2:** ALS criteria at inclusion according to site of onset.

	**Bulbar**	**Upper limb**	**Lower limb**	
***n***	**32**	**36**	**37**	
ALS-SSi	1.6 ± 1.4	0.7 ± 0.4	1.0 ± 0.9	ns
SVC loss (%)	1.4 ± 2.8	1.4 ± 2.5	2.4 ± 4.3	ns
Weight loss (kg)	0.5 ± 0.7	0.3 ± 1.1	−1.7 ± 11.2	ns
25OH vitamin D (ng/ml)	28.9 ± 11.3	25.8 ± 10.5	26.8 ± 19.9	ns

*Value are means ± SD*.

*ALS, amyotrophic lateral sclerosis; ALS-SSi, Revised Amyotrophic Lateral Sclerosis Functional Rating Scale severity score at inclusion; SVC, slow vital capacity; ns, not statistically significant*.

### Patients With the Lowest 25OH Vitamin D Levels Have the Worst Amyotrophic Lateral Sclerosis Prognosis

When 25OH VD was categorized into three groups (<15, >15 and <30, and >30 ng/ml), age of onset, SVC, and weight loss at inclusion were not significantly different between those three groups (Table 3). Conversely, patients with 25OH VD levels <15 ng/ml had significantly higher ALS-SSi than those with normal levels (>30 ng/ml), *p* = 0.011.

**Table 3:** ALS criteria at inclusion according to 25OH vitamin D levels.

**25OH vitamin D (ng/ml)**	***n***	**Age at onset**	**ALS-SSi**	**SVC**	**Weight loss**
<15	19	64.2 ± 9.7	1.20 ± 0.80	77.1 ± 21.2	0.074 ± 0.73
>15 and <30	47	61.1 ± 13.9	0.88 ± 0.72	83.6 ± 21.7	0.197 ± 0.51
>30	39	62.6 ± 10.9	0.75 ± 0.50	80.6 ± 23.3	0.363 ± 0.61
					
<15 vs. >30		ns	0.011	ns	ns

*ALS, amyotrophic lateral sclerosis; ALS-SSi, Revised Amyotrophic Lateral Sclerosis Functional Rating Scale severity score at inclusion; SVC, slow vital capacity; ns, not statistically significant*.

### 25OH Vitamin D Concentrations Are Independently Associated With ALS-SSe

**Figure 2:**
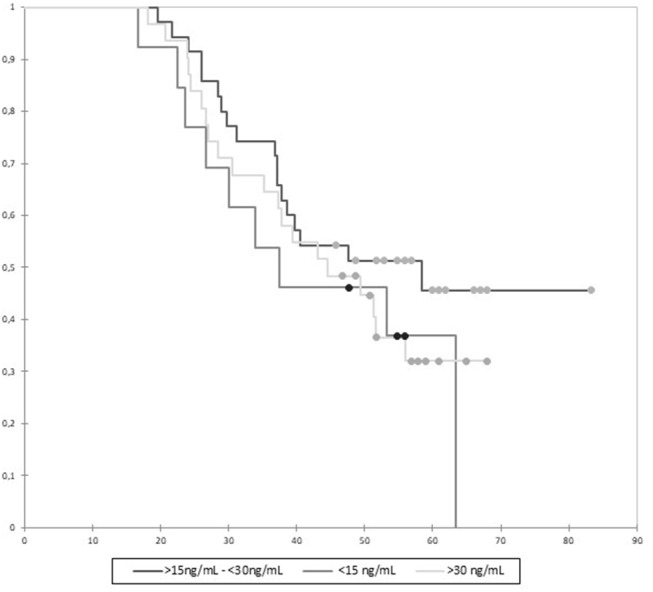
Kaplan–Meier analysis of survival according to 25OH vitamin D (VD) levels. Survival is expressed in months. Censored data are featured by full circles. Differences between groups were not significant (*p* = 0.88).

The influence of 25OH VD levels was studied further in the group of patients with at least 6 months' follow-up (*n* = 77). In this group, 62 had a 1 one-year follow-up, and 15 less than 1 year. The correlation between ALS-SSe and 25OH VD was studied using Spearman correlation analysis and did not show statistical significance: *r* = −0.197, *p* = 0.084. Conversely, univariate linear regression showed that ALS-SSe was associated with 25OH VD levels, ALS duration since onset, SVC at inclusion, and SVC loss (Table 4). The multivariate model found that 25OH VD levels were independently associated with ALS-SSe (*r* = −0.0125, *p* = 0.033), as were ALS duration at inclusion (*r* = −0.0281, *p* = 0.0074) and SVC at inclusion (*r* = −0.0101, *p* = 0.0037). Patients with the highest blood VD levels had a less severe ALS score of decline. The study of survival using log rank test with Kaplan–Meier curves did not show a significant difference of survival between the three groups defined by 25OH VD levels: <15, >15 and <30, and >30 ng/ml, *p* = 0.88 (Figure 2).

**Table 4:** Factors influencing ALS-SSe.

**Variables**	**Regression coefficient (SE)**	***p* value**	**Regression coefficient (SE)**	***p* value**
	**Univariate analysis**		**Multivariate analysis**	
25OH vitamin D	−0.0140 (0.0063)	*0.0291*	−0.0125 (0.0058)	*0.033*
Age of onset	0.0086 (0.0060)	*0.1591*		
ALS duration at inclusion	−0.0285 (0.0101)	*0.0062*	−0.0281 (0.0102)	*0.0074*
SVC at inclusion	−0.0110 (0.0036)	*0.0032*	−0.0101 (0.0034)	*0.0037*
Weight at inclusion	−0.0040 (0,0055)	*0.4713*		
SVC loss	0.1128 (0.0244)	*<0.0001*		
Weight loss	0.0044	*0.6631*		

## Discussion

This prospective study in ALS patients confirmed our previous results in a retrospective group: ALS-SSi, one important prognostic factor in the disease, was significantly higher in patients with severe 25OH VD deficiency (<15 ng/ml). Moreover, multivariate analysis of different prognostic factors of the disease showed that 25OH VD levels were an independent prognostic factor correlated to ALS-SSe. This is the first time such an influence is demonstrated in ALS. Previous works on 25OH VD influence gave conflicting results. However, methodologies were various, and the role of confounding factors on ALS-SS was not studied. Nevertheless, a series of biological studies have underlined the influence of VD on neuronal homeostasis and particularly of the motor neurons.

Our study is robust. The studied population well matched with classical ALS population. Some limitations, however, are to be underlined. The effect of 25OH VD deficiency on ALS-SS, in the present and prospective work, was of a lesser extent than our previous retrospective work on 74 ALS patients (7). Moreover, we did not here observe an influence of 25OH VD levels on survival. Apart from the possibility of recruitment biases in our previous, non-prospective, work, one potential reason for this difference could be the changes in medical practice. In our retrospective work, almost 30% of the ALS patients had severe VD deficiency, for example, below 15 ng/ml. In the present study, only 19 patients out of 105 (18%) had 25OH VD levels in that category. It is clear that physicians are now more aware of the importance of VD supplementation for patients' health, and this may explain the lower proportion of severely deficient patients. This is an important limit, and also for future works in the VD field. The influence of VD treatment on ALS natural history remains unknown. One important point in ALS would be to know whether the patients with VD supplementation have a better or worse prognosis. This point would be a key point as, if it was the case, it could mask the influence of severe VD deficiency in patients supplemented. Although prospective, one limit of the present work is the sample size of the population. This precludes really efficient and powerful subgroup analyses, as well as the study of a higher number of prognostic factors of ALS. The lower proportion of patients with severe VD deficiency nowadays would probably need to study very large samples.

One other potential limit to such a study on VD in ALS patients is that several factors, linked to daily life, may influence blood concentrations, such as food intake or sun exposure. Additionally, it has been suggested, in a retrospective analysis, that 25OH VD levels were correlated to gross motor function, suggesting that as handicap progresses, patients are limiting their outdoor activities and thus may have lower 25OH VD levels (9). Some of these points, linked to the concept of reverse causality, have been addressed in the present study. First, we examined relationships between 25OH VD levels and gross motor function, and no correlation could be found (not shown). Interestingly, in our retrospective work, patients with severe lower limb impairment were excluded, to avoid reverse causality, and when including them in a *post hoc* analysis, results were unchanged. Second, one parameter reflecting food intake in ALS patients is weight loss known as a prognostic marker of the disease. This parameter was included in the linear regression model with univariate and multivariate analyses; and weight loss, conversely to 25OH VD, was not independently associated with ALS-SSe. Other markers of nutrition such as albumin or prealbumin are interesting to study and more pertinent than weight loss, but they have not been included in this study, and this is a limit to take into account. Third, sun exposure was not introduced in our model for several reasons because, to our knowledge, seasonality has never been shown to influence any of the parameters of ALS. Moreover, seasonal variations of 25OH VD levels do exist but are usually modest, and a patient with severe VD deficiency is unlikely to have normal values a few months or seasons later without a supplementation. This element was corroborated in the present study in which at inclusion 25OH VD levels were similar between patients included in summer, spring, and autumn; only those recruited in winter had slightly lower levels (not shown). Aside seasonality, UV exposure may also modulate both VD levels and neuronal development/repair. This parameter is difficult to monitor, and one useful criterion has been used for multiple sclerosis: actinic degradation, which reflects efficiently past sun exposure. Such a pertinent parameter requires skin biopsies, and this was not performed in the present study.

Even though 25OH VD levels are independently associated with ALS-SSe in this prospective cohort, there are no strong arguments, to date, to propose a systematic VD supplementation in ALS, whatever the 25OH VD concentrations. If supplementation is required in severely deficient patients for medical reasons, there is no proof that this could influence ALS worsening in either way. It would be important that the present work be replicated in larger samples with multivariate analyses to confirm the important and independent prognostic roles of low 25OH VD levels, which we confirmed in this prospective study.

## Data Availability Statement

The datasets generated for this study are available on request to the corresponding author, without undue reservation, to any qualified researcher.

## Ethics Statement

The studies involving human participants were reviewed and approved by Ethics committee Sud-Méditerranée IV, CHU Saint Eloi, Montpellier, France. The patients/participants provided their written informed consent to participate in this study.

## Author Contributions

RJ-M, NP, FE, and WC recruited the patients and obtained patient consent. GM and MP were responsible for statistical analysis. A-MD was responsible for 25OH VD measurement. SA and LL acquired and managed data. WC wrote the manuscript. All authors critically reviewed the manuscript.

## Conflict of Interest

The authors declare that the research was conducted in the absence of any commercial or financial relationships that could be construed as a potential conflict of interest.
